# Popliteal Puzzle Solved: A Rare Case of Intramuscular Cysticercosis of the Peroneus Longus Muscle

**DOI:** 10.7759/cureus.102323

**Published:** 2026-01-26

**Authors:** Sameer K Mehta, Sandipan Mukhopadhyay, Shadab Shafi, Zaid M Nafe

**Affiliations:** 1 Medicine, Tata Main Hospital, Jamshedpur, IND; 2 Radiology, Tata Main Hospital, Jamshedpur, IND; 3 General Medicine, Tata Main Hospital, Jamshedpur, IND; 4 Gastroenterology, Tata Main Hospital, Jamshedpur, IND; 5 Community Medicine, Dr Vaishampayan Memorial Medical College, Solapur, IND

**Keywords:** intra-articular cysticercosis, knee cysticercosis, mri diagnosis, popliteal pain, taenia solium

## Abstract

Cysticercosis, caused by the larval form of *Taenia solium*, is endemic to many developing regions, with neurocysticercosis being the most recognized manifestation; however, musculoskeletal and isolated intra-muscular involvement is rare and diagnostically challenging. We report the case of a 37-year-old woman presenting with a two-week history of insidious popliteal pain in the left knee, initially evaluated by orthopedics and neurology with normal radiographs and nerve conduction studies. After referral to internal medicine, routine laboratory investigations and autoimmune markers were unremarkable, but MRI of the knee identified a well-defined cystic lesion with a central scolex, confirming intra-articular cysticercosis. The patient responded completely to oral albendazole and corticosteroids, with full symptomatic resolution and radiological normalization. This case highlights the importance of considering cysticercosis in the differential diagnosis of unexplained knee or soft-tissue pain, particularly in endemic areas; timely MRI evaluation facilitates accurate diagnosis, and conservative antiparasitic therapy can obviate the need for invasive interventions.

## Introduction

Cysticercosis is a parasitic infection caused by the larval form of *Taenia solium*, commonly acquired through ingestion of eggs via contaminated food or water. While it is classically associated with neurocysticercosis, where the central nervous system is involved, extra-neural manifestations such as muscular, ocular, and subcutaneous forms are also well recognized, albeit less common [1]. Musculoskeletal involvement is often asymptomatic and detected incidentally; however, in some cases, it may present with localized pain, swelling, or compressive symptoms that can mimic other orthopedic or neurological conditions [2,3].

Intramuscular cysticercosis around the knee is exceedingly rare. This form of presentation can be clinically deceptive, as patients typically undergo extensive evaluation by orthopedic surgeons or neurologists for complaints such as joint pain, swelling, or neuropathic features [4]. Popliteal fossa pain, in particular, often directs clinicians to consider differential diagnoses such as Baker’s cyst, deep vein thrombosis, meniscal injuries, or nerve entrapment syndromes [5]. However, parasitic causes such as cysticercosis are rarely considered in such anatomical regions, especially in the absence of systemic symptoms.

India is considered an endemic region for cysticercosis due to poor hygiene and sanitation [6]. Despite this, localized musculoskeletal presentations, especially involving the knee, remain underreported in clinical literature. MRI has emerged as the diagnostic modality of choice in such atypical cases, where it can reveal characteristic features such as well-defined cystic lesions with a central scolex, differentiating cysticercosis from other cystic or inflammatory lesions [7].

We report a rare and unusual case of popliteal pain secondary to knee joint cysticercosis in a middle-aged woman, initially evaluated by orthopedic and neurology specialists, ultimately diagnosed via MRI after physician referral. This case underscores the importance of maintaining a broad differential diagnosis in endemic regions and highlights the diagnostic value of imaging in resolving atypical presentations of parasitic infections.

## Case presentation

A 37-year-old woman presented to the outpatient department with complaints of persistent pain and discomfort in the posterior aspect of her left knee for the past two weeks. The pain was insidious in onset, dull aching in character, and aggravated by squatting or prolonged standing. There was no preceding history of trauma, fever, joint swelling, or systemic complaints such as weight loss or neurological symptoms. She denied any recent travel or exposure to tuberculosis.

**Figure 1:**
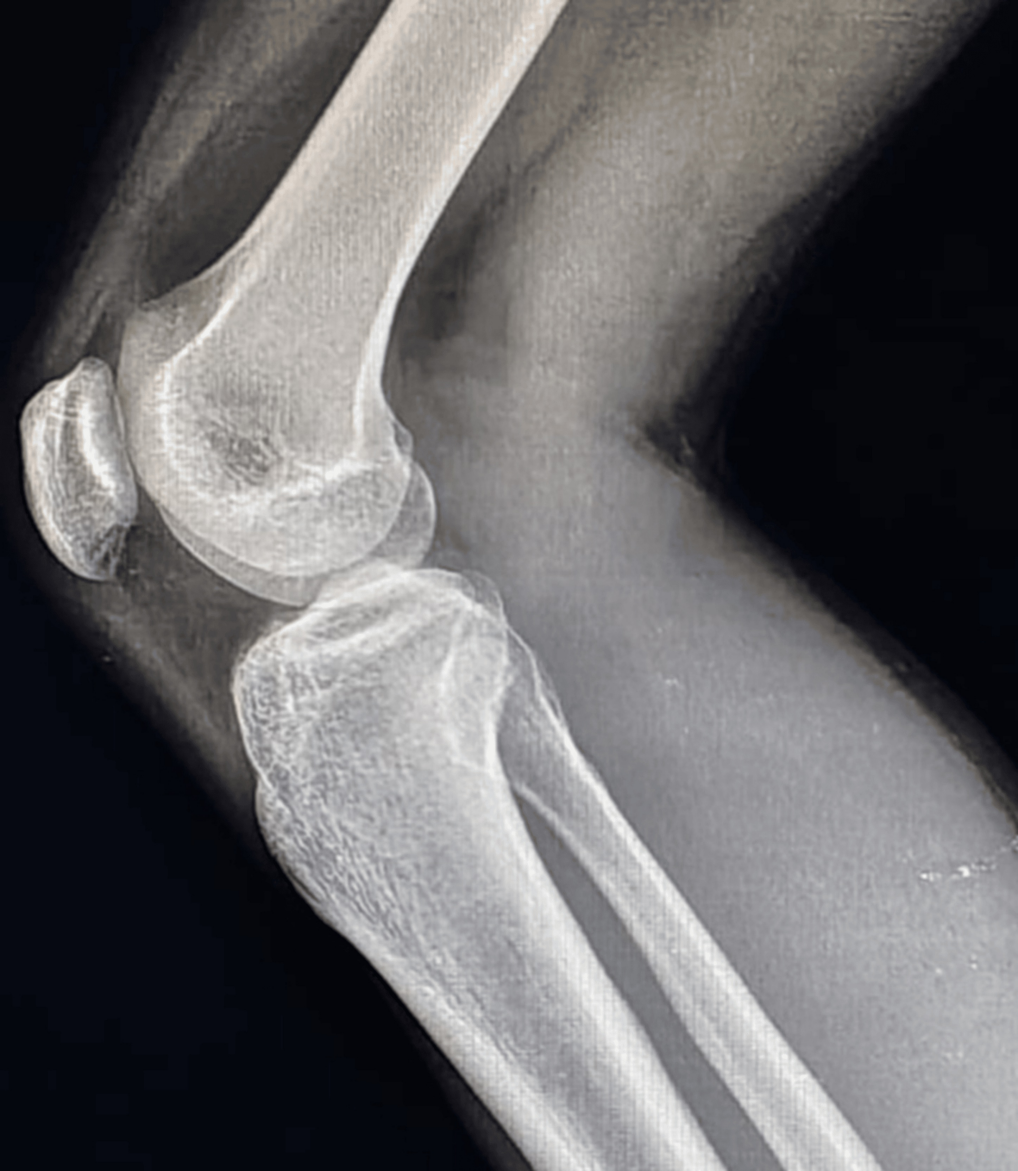
Plain radiograph of the knee with no intramuscular lesion appreciated in the lateral X-ray of the knee joint.

Initially, the patient sought orthopedic consultation, where a provisional diagnosis of early degenerative meniscal pathology or a Baker’s cyst was considered. A clinical examination revealed localized tenderness in the popliteal fossa without any visible swelling or signs of joint effusion. Range of motion was preserved, though terminal flexion elicited discomfort. There were no signs of ligamentous instability. A plain radiograph of the left knee did not reveal any bony abnormality or joint space narrowing (Figure 1).

She was subsequently referred to a neurologist due to radiation of pain toward the calf and concern for possible nerve entrapment or radiculopathy. Neurological examination was unremarkable, and no motor or sensory deficits were observed. Electromyography (EMG) and nerve conduction velocity (NCV) studies were normal, effectively ruling out peripheral nerve involvement or lumbosacral radiculopathy.

On re-evaluation by the internal medicine unit, a broader differential was considered. Routine blood investigations, including complete blood count (CBC), erythrocyte sedimentation rate (ESR), C-reactive protein (CRP), and fasting blood glucose, were within normal limits. Peripheral eosinophilia was not noted. Serological tests for rheumatoid factor, antinuclear antibodies (ANA), and anti-cyclic citrullinated peptide (anti-CCP) were negative, minimizing the likelihood of autoimmune arthritis.

**Figure 2:**
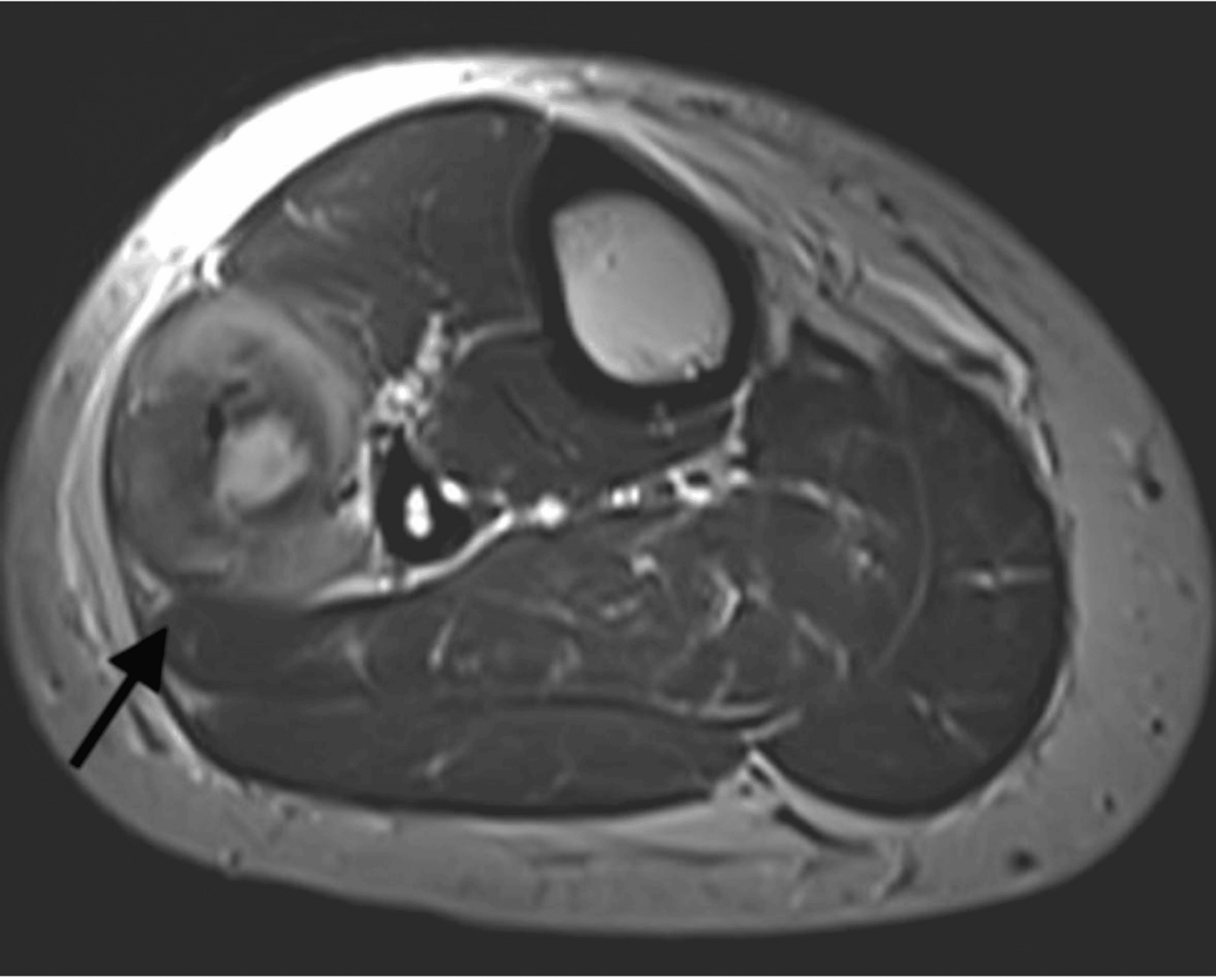
Axial T2-weighted MRI image of the leg showing a well-defined intramuscular lesion with surrounding bulky and inflamed peroneus muscle (black arrow). The lesion demonstrates an inhomogeneous hyperintense signal.

**Figure 3:**
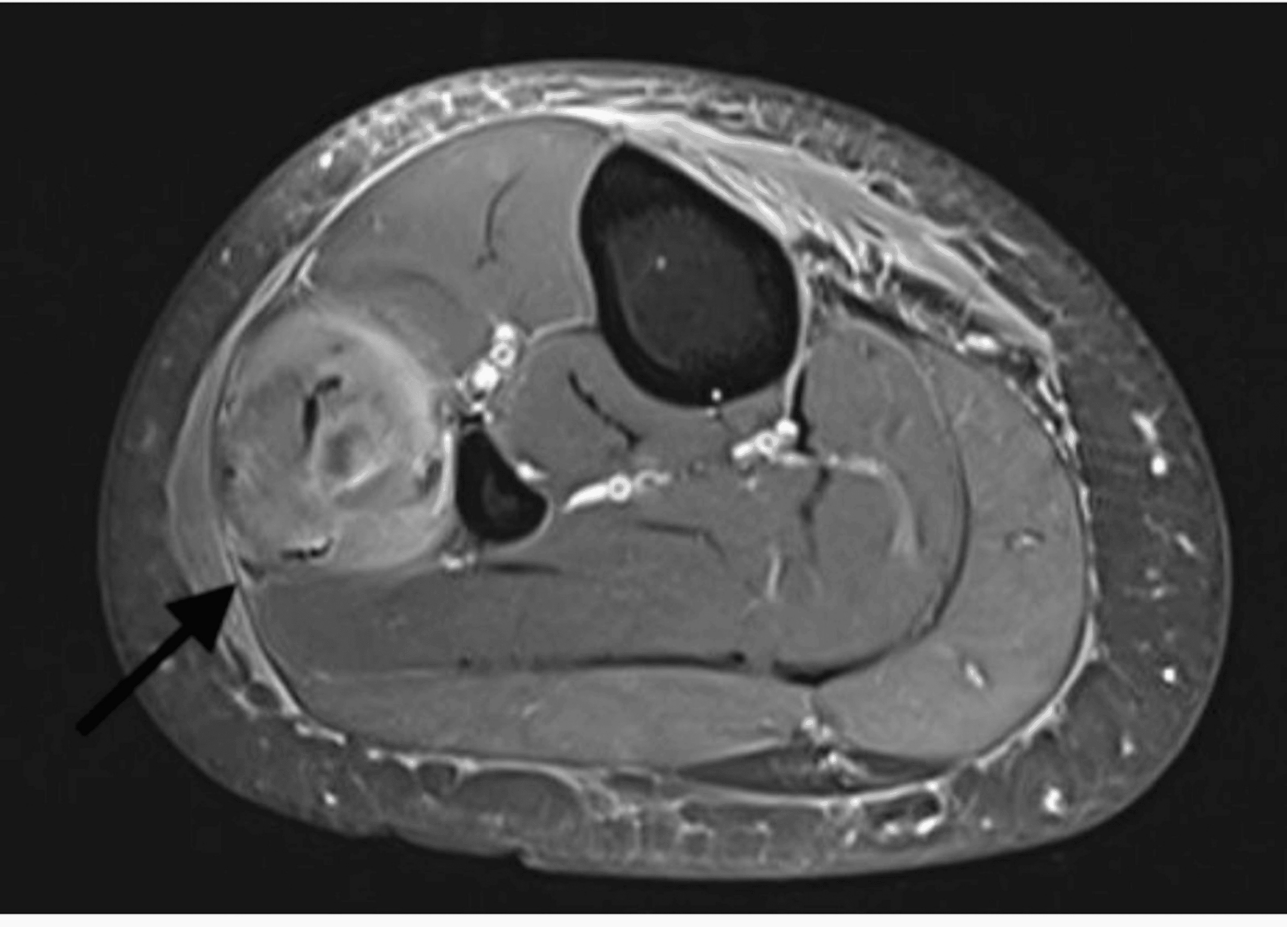
Axial post-contrast T1-weighted MRI image of the leg showing heterogeneously enhancing edematous and inflammatory peroneus muscle (black arrow) containing a hyperintense lesion.

**Figure 4:**
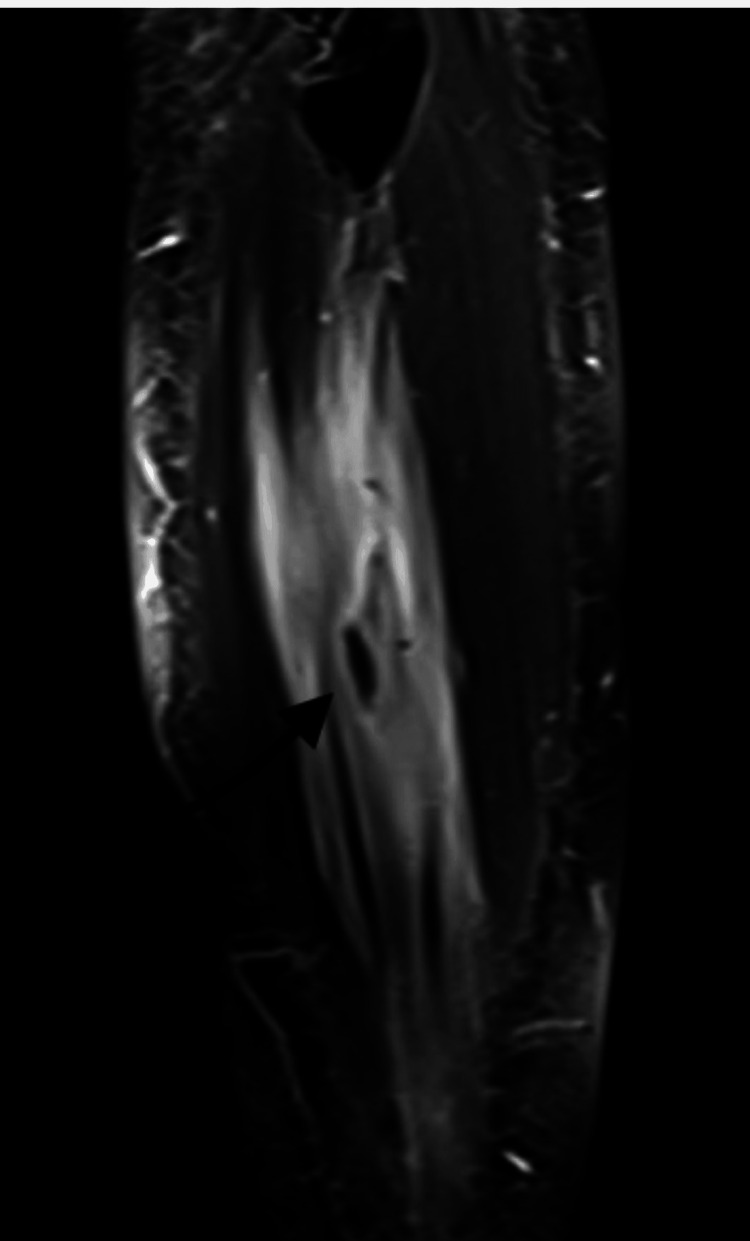
Coronal T2-weighted MRI image of the leg showing an intramuscular hypointense scolex (black arrow), characteristic of cysticercosis.

Given the persistent and unexplained nature of the popliteal pain, magnetic resonance imaging (MRI) of the left knee was advised. The MRI revealed a well-defined, oval-shaped cystic lesion measuring approximately 1.4 × 1.2 cm located in the lateral compartment of the leg within the peroneus longus muscle. The lesion appeared hypointense on T1-weighted images and hyperintense on T2-weighted images, with a central eccentric dot-representing the scolex. These imaging findings were consistent with a diagnosis of isolated intramuscular cysticercosis of leg (Figures 2-4).

**Figure 5:**
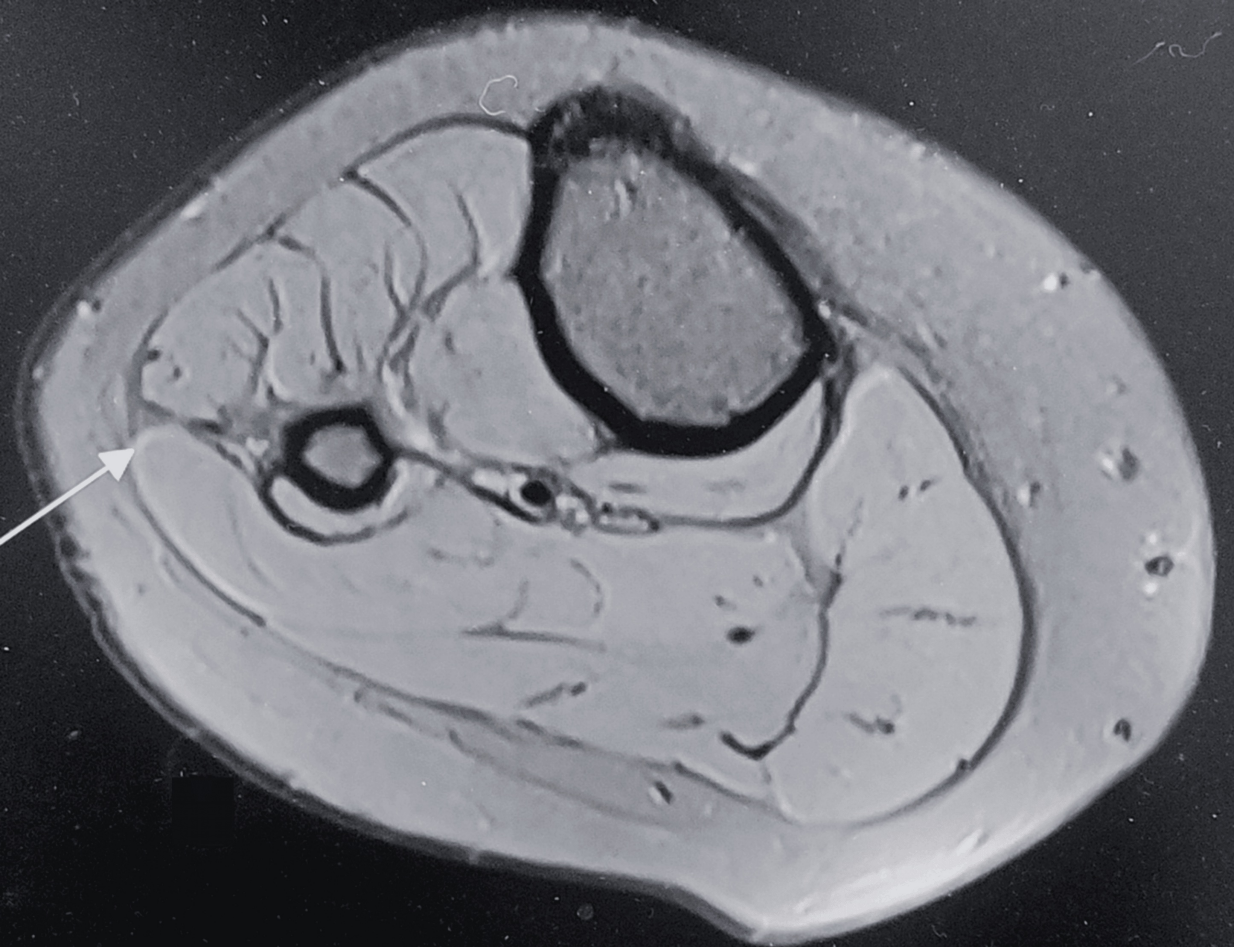
Post-treatment follow-up axial PD FSE image revealing complete resolution of the disease PD FSE: Proton density-weighted fast spin echo MRI

The patient was started on oral albendazole 15 mg/kg/day in two divided doses for 28 days, along with oral corticosteroids (prednisolone 1 mg/kg/day) tapered over two weeks to reduce inflammatory response due to larval degeneration. The patient was also advised to rest, take analgesics for symptomatic relief, and undergo periodic follow-up. At six weeks post-treatment, the patient reported complete resolution of symptoms with no recurrence of pain. A repeat clinical examination and follow-up MRI of the leg revealed complete regression of the lesion (Figure 5).

## Discussion

Cysticercosis is a parasitic infestation caused by the larval stage of *Taenia solium*, predominantly affecting individuals in regions with poor sanitation and high prevalence of porcine contact. While neurocysticercosis remains the most commonly recognized clinical manifestation, extra-neural forms such as subcutaneous, muscular, ocular, and even cardiac cysticercosis have been increasingly reported in endemic areas like India [1,2]. Musculoskeletal involvement accounts for approximately 1%-2% of all cysticercosis cases and is often asymptomatic, discovered incidentally during imaging or surgery [3].

Isolated intramuscular cysticercosis of the leg, particularly within or near the popliteal region, is extremely rare. In most instances, such a presentation leads to a diagnostic dilemma due to its nonspecific symptoms, including localized pain, swelling, and restricted joint movements. These symptoms often mimic more common conditions such as Baker’s cyst, bursitis, tubercular abscess or nerve entrapment syndromes [4]. In our case, the patient underwent orthopedic and neurological consultations before being referred for a detailed evaluation by a physician, which highlights the diagnostic challenge posed by this rare entity.

Initial investigations including plain radiographs and laboratory parameters are typically non-contributory in such cases, as seen in this patient. The absence of eosinophilia and normal inflammatory markers does not exclude the diagnosis. EMG-NCV studies are also not useful unless neural involvement is suspected. Thus, MRI becomes the imaging modality of choice, providing both anatomical detail and lesion characterization. The hallmark MRI finding suggestive of cysticercosis is a well-defined cystic lesion with an eccentric hypointense scolex-best appreciated on T2-weighted sequences [5-7].

The presence of such imaging features in our patient confirmed the diagnosis of isolated soft tissue cysticercosis near the knee joint. Although ultrasound and CT scans may also detect cystic lesions, MRI remains superior in soft tissue differentiation and scolex visualization, which is crucial for diagnosis [8,9]. Cases involving the masseter, thigh, and biceps muscles have been previously described, but periarticular cysticercosis, especially in the popliteal fossa, remains underreported in the literature [10-12].

Management includes antiparasitic therapy with albendazole or praziquantel, along with steroids to attenuate inflammatory reactions resulting from larval death. In our patient, treatment with albendazole and a tapering course of corticosteroids led to complete clinical and radiological resolution. Surgical excision is generally reserved for lesions causing mechanical obstruction, persistent pain, or diagnostic uncertainty [13].

This case underscores the importance of considering cysticercosis in the differential diagnosis of chronic monoarticular pain or soft tissue swellings in endemic settings. Early MRI imaging and a high index of suspicion are key to preventing unnecessary interventions and guiding appropriate therapy. Clinicians across specialties should be aware of this atypical presentation to avoid misdiagnosis.

## Conclusions

Intramuscular cysticercosis of the leg is an exceedingly rare manifestation of a common parasitic infection, particularly when it presents as isolated popliteal pain. This case illustrates the diagnostic challenges posed by atypical presentations and highlights the importance of maintaining a high index of suspicion in endemic areas. MRI played a pivotal role in establishing the diagnosis, showing characteristic features of a cysticercus cyst with a central scolex. Prompt initiation of antiparasitic therapy combined with corticosteroids resulted in complete clinical recovery, avoiding unnecessary surgical intervention. Awareness of such unusual presentations among clinicians across specialties can facilitate timely diagnosis and appropriate management, preventing misdiagnosis and overtreatment.
